# Retinal progenitor cells (jCell) for retinitis pigmentosa

**DOI:** 10.3389/fncel.2025.1646156

**Published:** 2025-08-25

**Authors:** Jing Yang, Baruch D. Kuppermann, David Liao, Mitul C. Mehta, Chinhui Hsiang, Steven Menges, David S. Boyer, Henry Klassen

**Affiliations:** ^1^Stem Cell Research Center, University of California, Irvine, Irvine, CA, United States; ^2^Gavin Herbert Eye Institute, University of California, Irvine, Irvine, CA, United States; ^3^Retina-Vitreous Associates Medical Group, Los Angeles, CA, United States

**Keywords:** intravitreal, cell therapy, allogeneic transplantation, photoreceptor dystrophy, blindness

## Abstract

**Objective:**

To assess the safety and tolerability of intravitreal injection of human retinal progenitor cells (RPCs) at multiple dose levels in adults with non-syndromic retinitis pigmentosa (RP).

**Design:**

A prospective, multicenter, open-label, single-arm, Phase I/IIa safety study of RPCs in adults with RP (*n* = 28). Two patient cohorts were studied: Cohort 1: BCVA no better than 20/200 and no worse than Hand Motions, and Cohort 2: BCVA no better than 20/40 and no worse than 20/200).

**Subjects:**

Adults (*n* = 28) with a clinical diagnosis of RP confirmed by electroretinogram, consenting to gene mutation typing for genes involved in inherited retinal degenerations and related disorders, and willing to undergo human leukocyte antigen (HLA) typing.

**Methods:**

Subjects, who were not selected for genotype, were divided across the two vision cohorts with each receiving a single intravitreal injection of one of: 0.5, 1.0, 2.0, or 3.0 × 10^6^ allogeneic RPCs. Initially, subjects received the lowest dose (0.5 × 10^6^ RPCs) in the worse-seeing eye. Each dose group contained equal numbers of subjects from Cohorts 1 and 2.

**Results:**

Intravitreal RPC injection was well tolerated and associated with mostly transient mild to moderate adverse events. There were no signs of graft rejection. While primarily a safety study, exploratory efficacy assessments suggested improved BCVA measurements at all doses, with a possible dose-response at the highest levels. Mean BCVA change from pre-treatment to Month 12 in the treated vs untreated eyes was 1.4 letters for the 0.5 × 10^6^ dose group, 1.0 letters for the 1.0 × 10^6^ group, 4.8 letters for the 2.0 × 10^6^ group, and 9.0 letters for the 3.0 × 10^6^ group. Additional patient-reported changes included increased light sensitivity, improved object recognition, color discrimination, and reading.

**Conclusion:**

A single intravitreal injection of RPCs was well tolerated in this safety study. The exploratory efficacy data suggest potential improvement of BCVA in some RP patients, particularly at the highest dose. While viewed cautiously, the possible treatment effect should be further investigated in larger controlled studies. The RPC technology has received FDA Regenerative Medicine Advanced Therapy designation. Later phase studies are ongoing.

**Clinical trial registration:**

https://clinicaltrials.gov/study/NCT02320812, NCT02320812.

## 1 Introduction

Retinitis pigmentosa (RP) is an inherited neurodegenerative retinopathy caused by the loss of photoreceptors and usually characterized by retinal pigment deposits visible on fundus examination (Hamel, 2006). Archetypically, RP presentation involves a primary degeneration of the rod photoreceptors, with secondary degeneration of cones, and may be described as a rod-cone dystrophy.

Retinitis pigmentosa (RP) leads to retinal degeneration with vision and visual field loss (Hamel, 2006). As rod photoreceptors provide vision at low illumination, the initial symptom of RP is night blindness, which is often ignored by patients in early disease stages (Campochiaro et al., 2020; Hamel, 2006). As such, diagnosis is difficult to establish at this stage, particularly in the 50% of cases without familial history (Hamel, 2006).

Night blindness is followed by progressive loss of the peripheral visual field during daylight, impairment of visual functioning, and photophobia (especially in diffuse light) (Hamel, 2006). Night blindness is associated with debilitating difficulties in spatial orientation necessary for driving and general mobility (Azoulay et al., 2015; Campochiaro et al., 2020). Following the loss of rods, the cones of the macula are affected with the central island of functional cones becoming progressively smaller, resulting in visual acuity loss and severe disability as a later disease feature (Campochiaro et al., 2020).

Retinitis pigmentosa (RP) is a relentlessly progressive condition leading to incurable legal blindness; photoreceptors do not regenerate once lost. Genetically, it is highly heterogeneous with more than 90 genes linked to RP (Nguyen et al., 2023). While some extreme cases are reported with a rapid evolution over 20 years, gradual visual degeneration usually occurs over several decades (Hamel, 2006). The prevalence of RP in the United States and Europe is approximately 1 in 3,500 (Fahim et al., 2023).

jCell is a live suspension of allogeneic retinal progenitor cells (RPCs), originally derived from fetal tissue. The characteristics and functional properties of these RPCs have been extensively evaluated *in vitro* and in animal models (Yang et al., 2024). RPCs delivered by intravitreal injection do not replace host photoreceptor cells, but appear to exert a diffusible trophic effect. The cells release a range of factors with potential neurotrophic and/or neuroprotectant activity (Yang et al., 2024).

The factors include PEDF (Steele et al., 1993), humanin (Hashimoto et al., 2001; Solanki et al., 2019), MANF (Neves et al., 2016), basic fibroblast growth factor (bFGF) (Faktorovich et al., 1990), Osteopontin (OPN) (Del Rio et al., 2011), the Midkine family member pleiotrophin (Furuta et al., 2004), and Midkine itself (Unoki et al., 1994), These are associated with a range of effects including rescue of photoreceptors (Del Rio et al., 2011; Unoki et al., 1994) and retinal ganglion cells (Duan et al., 2015), reduction of oxidative stress (Sreekumar et al., 2016), and reduction of retinal endoplasmic reticulum stress (Gao et al., 2025).

Retinal progenitor cells RPCs have low/nil expression levels of MHC Class II antigens (Yang et al., 2024) and are well tolerated as allografts in the vitreous cavity, without systemic immune suppression, and survive for a prolonged period (Yang et al., 2024).

Preclinical studies in a model of rod-cone dystrophy (the RCS rat) demonstrate that intravitreal injection of RPCs results in preservation of photoreceptors and the outer plexiform layer, as well as the amelioration of functional deficits (Yang et al., 2024). Positive effects were also observed on non-neuronal cells with relative normalization of the morphology of the RPE and relative normalization of gene expression levels in Mueller cells and astrocytes (Yang et al., 2024).

Further work in a human cell line from patients with retinal degeneration (AMD cybrids) shows that RPCs increase cell viability and decrease gene expression related to apoptosis, autophagy, and endoplasmic reticulum stress compared with cybrids without RPC treatment (Yu et al., 2021).

Preclinical proof-of-principle data and formal toxicology study results contributed to the decision to file an IND with the FDA and conduct the initial clinical trial described herein.

RPCs are being explored clinically for use as a genetically agnostic treatment for RP. The primary goal of RPC therapy is to preserve, and potentially improve, vision by intervening at a time when dystrophic host photoreceptors can be protected and potentially reactivated. jCell delivery is via conventional intravitreal injection under local anesthesia in an outpatient setting.

The primary objective of this study was to evaluate the safety of jCell injection in adult patients with non-syndromic RP. The secondary objective was to evaluate potential therapeutic response in visual function over a 12-month period following a single RPC injection.

## 2 Methods

This was a prospective, multicenter, open-label, single-arm, Phase I/IIa trial of human retinal progenitor cells (jCell) in patients with a clinical diagnosis of RP confirmed by electroretinogram (ERG). The research protocol was approved by relevant institutional review boards or ethics committees and all human participants gave written informed consent. The study was conducted in compliance with the study protocol, Good Clinical Practice according to the International Conference on Harmonization guidelines, and ethical principles that are consistent with the Declaration of Helsinki.

Cells used in the trial were manufactured in the GMP Facility within the Institute for Regenerative Cures at UC Davis Medical Center. Briefly, donated GTP tissue underwent trituration and dissociation into component cells which were cultured under standard normoxic conditions (37°C, 5% CO_2_) to low passage number prior to final harvest. Pooled cells were aliquoted into cryovials prior to freezing and storage in liquid nitrogen. Samples from each manufactured lot underwent testing and met preestablished criteria for cell count, viability, marker expression, karyotype and colony formation (Yang et al., 2024). Prior to use, individual vials were thawed and the cells cultured to reestablish metabolic activity, then harvested, washed, and resuspended in saline (BSS +) prior to injection. Each prepared dose was gram stained and tested for cell count, viability, endotoxin, sterility and mycoplasma.

Study subjects were screened for eligibility and informed consent obtained; a total of 28 patients was planned for study inclusion. Study subjects, who were not selected for genotype, were divided equally across the two vision cohorts (*n* = 14), with each patient receiving one of four doses of jCell: a live suspension of either 0.5, 1.0, 2.0, or 3.0 × 10^6^ allogeneic human RPCs as an intravitreal injection (50 μL volume) under local anesthesia: 8 subjects (4 from Cohort 1 and 4 from Cohort 2) received 0.5 × 10^6^ RPCs; 8 subjects (4 from Cohort 1 and 4 from Cohort 2) received 1.0 × 10^6^ RPCs; 6 subjects (3 from Cohort 1 and 3 from Cohort 2) received 2.0 × 10^6^ RPCs; and 6 subjects (3 from Cohort 1 and 3 from Cohort 2) received 3.0 × 10^6^ RPCs. The RPCs used in the clinical program were characterized using techniques including microarray transcriptome analysis, since the gene expression profile of RPCs can be distinguished from other cell types, protein expression characteristic of proliferating progenitor cells, and low levels of expression of MHC Class II antigens. The HLA type of each lot of RPCs was also established so that if a new HLA antibody was noted in any subject, it could be assessed in the context of the HLA profile of the subject and the injected cells.

Apart from cell preparation, each RPC injection procedure took approximately 2–5 min to complete and was similar to routine office-based intravitreal injection. Subjects enrolled initially received the lowest dose (0.5 × 10^6^ RPCs) in the eye with poorest vision.

The primary study objective was to assess the safety and tolerability of an RPC injection at multiple dose levels in adult subjects with non-syndromic RP. Safety and tolerability were assessed on an ongoing basis by evaluation of adverse events, identification of any dose-limiting toxicities, physical examinations and vital signs, clinical laboratory values, and anti-drug antibodies. In addition, ophthalmic safety assessments included anterior and posterior ocular examination, macular SD-OCT, B-scan, and intraocular pressure (IOP) monitoring. Vitreous examination and B-scans were also utilized to assess the appearance of the injected cells at different time points during the study. To reduce the risk of intraocular inflammation after the procedure, subjects were treated with corticosteroid eye drops for up to 14 days, including a taper schedule in the second week at the discretion of the investigator. No systemic immune suppression was used. Following RPC administration to the first subject, there was a minimum 4-week interval to confirm no serious injection-related adverse events occurred prior to treatment of the second subject.

The secondary study objective was to monitor ocular function over a 12-month period following a single intravitreal jCell injection to determine the potential therapeutic response in subjects with non-syndromic RP. Therapeutic response was primarily assessed with measurements of best corrected visual acuity (BCVA). Although this study was designed as a single arm, uncontrolled study, both treated and untreated eyes were monitored for changes in vision over time, with the untreated eye serving as an informal control that might provide useful background information with respect to deterioration of vision in untreated eyes. Visual Fields were also assessed. Macular spectral domain optical coherence tomography (SD-OCT) and autofluorescence were also performed and analyzed to determine any notable progression of imaging abnormalities in the treated eyes during the study period.

ERG testing was performed at baseline, 6-, and 12-month study visits and measurements were obtained for a-wave amplitude, b-wave amplitude, time from flash onset to a wave trough, and time from flash onset to b-wave peak.

To assess a broader range of doses, provide an opportunity to observe a potential dose-response-effect in ophthalmology assessments, and facilitate the planning of future studies, a key protocol amendment was implemented following administration of the initial two dose levels. The number of dose levels tested was increased from two (0.5 × 10^6^ and 1.0 × 10^6^ RPC) to four (0.5, 1.0, 2.0 and 3.0 × 10^6^ RPC) for each cohort, with four subjects at each of the first two dose levels, and three subjects at each of the higher two dose levels.

### 2.1 Demography

Adult patients (> 18 years) were included if presenting with a clinical diagnosis of RP confirmed by ERG, consenting to gene mutation typing for eye disease-related genes known to be involved in inherited retinal degenerations and related disorders (if unavailable from prior testing), and willing to provide a blood sample for human leukocyte antigen (HLA) typing (if unavailable from prior testing). Gene mutation types were not used as inclusion or exclusion criteria.

Two patient cohorts were studied: subjects classified as legally blind based on baseline BCVA (Cohort 1: BCVA no better than 20/200 and no worse than Hand Motions), and those with less marked BCVA loss (Cohort 2: BCVA no better than 20/40 and no worse than 20/200). Subjects received a single intravitreal dose of RPC on study Day 0 in the eye with the poorest vision and were followed for 1 year post-injection, with a separate study planned to follow-up patients for an additional 2 years following study completion.

### 2.2 Statistical methods

The intention-to-treat (ITT) population was defined as all subjects enrolled in the study and who provided any post-screening data. The safety population was defined as all subjects who received jCell treatment.

As primarily an exploratory safety study, no formal hypothesis testing occurred and data were summarized for all subjects, with descriptive statistics used to tabulate and summarize study outcomes. The baseline results of clinical examinations of the injected eye were used as controls, while data from non-treated eyes were also assessed. For the primary safety outcome, adverse events were monitored by the investigator and the subject and summary analyses performed using descriptive statistics. For the exploratory efficacy analyses, continuous variables were summarized descriptively and discrete variables summarized by frequency or percentage. Each individual subject’s fellow eye was used as the control.

## 3 Results

A total of 28 patients with a clinical diagnosis of RP were included. The mean age of subjects was 49.2 years (range 18–73), with 60.7% female and 39.5% male. Most patients were white (85.7%) and were not Hispanic or Latino (75%). Subjects were assigned to either Cohort 1 (*n* = 14) or Cohort 2 (*n* = 14) (Table 1). In each cohort, patients received a single intravitreal injection at doses of 0.5 × 10^6^ RPC (*n* = 4), 1.0 × 10^6^ RPC (*n* = 4), 2.0 × 10^6^ RPC (*n* = 3), or 3.0 × 10^6^ RPC (*n* = 3). Exactly half of patients had their right eye designated as the study eye. All patients were included in the ITT and safety population; all enrolled patients completed the study.

**TABLE 1:** Baseline patient-specific characteristics.

	Subject	RPCs* dose	Cohort	Study eye	Age	Sex	Race	Ethnicity	Causative gene	Lens status	Vitreous status
001–003	1	0.5 × 10^6^	1	OS	64	Female	White	Hispanic or Latino	NR2E3	Pseudophakic	Vitreous condensations, mild + 1
001–004	2	0.5 × 10^6^	1	OD	51	Male	White	Hispanic or Latino	RPGR	Phakic	Normal
002–001	3	0.5 × 10^6^	1	OS	58	Female	White	Not Hispanic or Latino	C2orf71	Phakic	Normal
002–002	4	0.5 × 10^6^	1	OD	28	Female	Asian	Not Hispanic or Latino	Indeterminate	Phakic	Normal
001–010	5	0.5 × 10^6^	2	OS	69	Female	White	Not Hispanic or Latino	Indeterminate	Pseudophakic	PVD,** mild + 1
001–011	6	0.5 × 10^6^	2	OD	38	Male	White	Not Hispanic or Latino	IFT140	Phakic	PVD, Syneresis, moderate + 2
001–012	7	0.5 × 10^6^	2	OD	57	Female	White	Not Hispanic or Latino	IMPG2	Phakic	PVD, syneresis, mild + 1
002–009	8	0.5 × 10^6^	2	OS	18	Male	White	Not Hispanic or Latino	Indeterminate	Phakic	Normal
001–005	9	1.0 × 10^6^	1	OD	36	Female	White	Not Hispanic or Latino	Indeterminate	Phakic	Syneresis, moderate + 2
002–007	10	1.0 × 10^6^	1	OD	59	Male	White	Not Hispanic or Latino	Indeterminate	Pseudophakic	Normal
001–006	11	1.0 × 10^6^	1	OS	45	Female	White	Hispanic or Latino	Indeterminate	Phakic	PVD, mild + 1
002–008	12	1.0 × 10^6^	1	OD	40	Male	White	Not Hispanic or Latino	CLRN1	Pseudophakic	PVD, mild + 1
002–013	13	1.0 × 10^6^	2	OS	52	Female	White	Hispanic or Latino	RP1	Pseudophakic	Normal
002–014	14	1.0 × 10^6^	2	OS	30	Male	White	Not Hispanic or Latino	ABCC6	Phakic	PVD, mild + 1
002–015	15	1.0 × 10^6^	2	OS	60	Male	White	Not Hispanic or Latino	USH2A	Pseudophakic	PVD, mild + 1
002–016	16	1.0 × 10^6^	2	OD	56	Male	White	Not Hispanic or Latino	RHO	Pseudophakic	PVD, mild + 1
001–202	17	2.0 × 10^6^	1	OD	72	Female	White	Not Hispanic or Latino	Indeterminate	Pseudophakic	Partial PVD, mild + 1
002–203	18	2.0 × 10^6^	1	OS	29	Female	White	Hispanic or Latino	CRB1	Phakic	Anterior vitreous cells, mild + 1
002–201	19	2.0 × 10^6^	1	OD	62	Male	White	Not Hispanic or Latino	CERKL	Phakic	PVD, Mild + 1
001–207	20	2.0 × 10^6^	2	OD	42	Male	White	Hispanic or Latino	RP2	Phakic	Syneresis, mild + 1
001–208	21	2.0 × 10^6^	2	OD	73	Female	White	Not Hispanic or Latino	EYS	Pseudophakic	Syneresis, floaters, PVD, mild + 1
001–209	22	2.0 × 10^6^	2	OS	54	Female	White	Not Hispanic or Latino	Indeterminate	Pseudophakic	Clear vitreomacular adhesion, mild + 1
001–204	23	3.0 × 10^6^	1	OD	48	Female	Asian	Not Hispanic or Latino	EYS, CNGB1	Phakic	Syneresis, mild + 1
002–206	24	3.0 × 10^6^	1	OS	54	Female	Asian	Not Hispanic or Latino	PRPF8	Pseudophakic	PVD, mild + 1
001–205	25	3.0 × 10^6^	1	OD	67	Female	White	Not Hispanic or Latino	MT-ATP6	Phakic	Syneresis, mild + 1
001–211	26	3.0 × 10^6^	2	OS	48	Female	White	Hispanic or Latino	Indeterminate	Pseudophakic	PVD, mild + 1
002–210	27	3.0 × 10^6^	2	OS	28	Male	White	Not Hispanic or Latino	USH2A	Phakic	PVD, mild + 1
002–212	28	3.0 × 10^6^	2	OS	40	Female	Asian	Not Hispanic or Latino	CLRN1, RP1	Pseudophakic	PVD, mild + 1

*RPCs, retinal progenitor cells;

**PVD, posterior vitreous detachment.

For the primary safety analysis, a single intravitreal injection of up to 3.0 × 10^6^ RPC was considered well tolerated with mostly transient, low grade adverse events reported. No subject was discontinued due to an adverse event. In total, 89.3% of patients experienced at least one treatment-emergent adverse event, with approximately half associated with eye disorders (45/99). Adverse events considered as related to study treatment occurred in all dose groups and both cohorts but did not appear to correlate with either dose level or vision cohort. All ocular treatment-emergent adverse events are shown in Table 2.

**TABLE 2:** Subjects (% of dose group) experiencing ocular treatment emergent adverse events (TEAEs) by preferred term and maximum severity (as defined by the common terminology criteria for adverse events developed by the U.S. National Cancer Institute).

	0.5 × 10^6^ RPCs			1.0 × 10^6^ RPCs			2.0 × 10^6^ RPCs			3.0 × 10^6^ RPCs			Total		
	(*N* = 8)			(*N* = 8)			(*N* = 6)			(*N* = 6)			(*N* = 28)		
System organ class/ preferred term	Mild	Moderate	Severe	Mild	Moderate	Severe	Mild	Moderate	Severe	Mild	Moderate	Severe	Mild	Moderate	Severe
Eye disorders	6 (75.0%)	0	0	7 (87.5%)	0	0	3 (50.0%)	2 (33.3%)	0	4 (66.7%)	0	0	20 (71.4%)	2 (7.1%)	0
Anterior chamber cell	0	0	0	0	0	0	0	1 (16.7%)	0	0	0	0	0	1 (3.6%)	0
Anterior chamber disorder	0	0	0	0	0	0	1 (16.7%)	0	0	0	0	0	1 (3.6%)	0	0
Anterior chamber flare	1 (12.5%)	0	0	2 (25.0%)	0	0	0	0	0	2 (33.3%)	0	0	5 (17.9%)	0	0
Anterior chamber pigmentation	0	0	0	1 (12.5%)	0	0	1 (16.7%)	0	0	1 (16.7%)	0	0	3 (10.7%)	0	0
Cataract	1 (12.5%)	0	0	0	0	0	0	0	0	0	0	0	1 (3.6%)	0	0
Cataract nuclear	0	0	0	0	0	0	1 (16.7%)	0	0	0	0	0	1 (3.6%)	0	0
Conjunctival hemorrhage	2 (25.0%)	0	0	5 (62.5%)	0	0	2 (33.3%)	0	0	1 (16.7%)	0	0	10 (35.7%)	0	0
Corneal pigmentation	0	0	0	0	0	0	1 (16.7%)	0	0	0	0	0	1 (3.6%)	0	0
Eye inflammation	0	0	0	0	0	0	0	0	0	1 (16.7%)	0	0	1 (3.6%)	0	0
Eye pain	2 (25.0%)	0	0	1 (12.5%)	0	0	1 (16.7%)	0	0	1 (16.7%)	0	0	5 (17.9%)	0	0
Eyelids pruritus	0	0	0	0	0	0	1 (16.7%)	0	0	0	0	0	1 (3.6%)	0	0
Iris adhesions	0	0	0	0	0	0	0	1 (16.7%)	0	0	0	0	0	1 (3.6%)	0
Iritis	0	0	0	0	0	0	0	1 (16.7%)	0	0	0	0	0	1 (3.6%)	0
Keratic precipitates	0	0	0	0	0	0	1 (16.7%)	0	0	0	0	0	1 (3.6%)	0	0
Lenticular opacities	0	0	0	0	0	0	1 (16.7%)	0	0	0	0	0	1 (3.6%)	0	0
Ocular discomfort	0	0	0	1 (12.5%)	0	0	0	0	0	0	0	0	1 (3.6%)	0	0
Posterior capsule opacification	0	0	0	0	0	0	0	1 (16.7%)	0	0	0	0	0	1 (3.6%)	0
Vision blurred	0	0	0	0	0	0	1 (16.7%)	0	0	0	0	0	1 (3.6%)	0	0
Visual impairment	0	0	0	0	0	0	1 (16.7%)	0	0	0	0	0	1 (3.6%)	0	0
Vitreal cells	0	0	0	0	0	0	1 (16.7%)	0	0	0	0	0	1 (3.6%)	0	0
Vitreous floaters	0	0	0	0	0	0	1 (16.7%)	0	0	0	0	0	1 (3.6%)	0	0

For most patients, adverse events were considered mild (71.4%) or moderate (14.3%). Only one subject experienced a severe (Grade 3) event of cells in the anterior chamber, which was considered as expected considering the RPC injection. There were four Data Monitoring Committee reviews of safety data, with no important safety concerns noted at any time point.

There was a single serious adverse event of arthralgia reported initially as possibly related to study treatment in Cohort 1 (2.0 × 10^6^ RPC). Subsequent investigation determined the event relationship as unlikely and associated with an autoimmune event.

**FIGURE 1:**
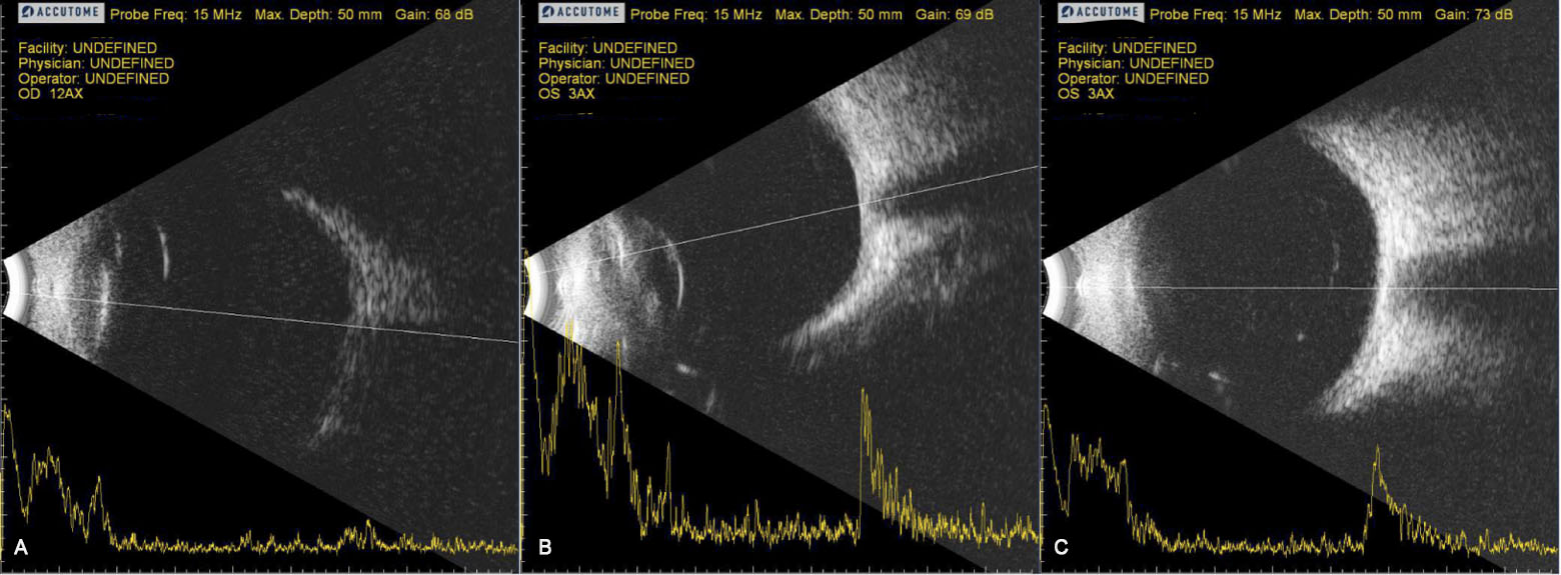
Representative B-scans 7 days, 6 months, and 12 months after jCell injection. **(A)** 7 days post injection. **(B)** 6 Months post injection. **(C)** 12 Months post injection.

Patients were also monitored for safety using a variety of ocular tests, including OCT to monitor for cystoid macular edema (Table 3), IOP, slit lamp and fundus exams, fluorescence angiography, autofluorescence, and B-scans (data not shown). Injected RPCs tended to coalesce into clusters within the vitreous cavity and were typically visible on examination as a small spherule of white cells, often evolving a net-like, reticular architecture over time, and sometimes dispersing into sheets. Injected cells typically localized to the inferotemporal and inferior vitreous, out of the visual axis. Figures 1A–C are representative of B-scan appearance 7 days, 6 months, and 12 months after jCell injection. Cells remained visible on examination for variable lengths of time; up to 12 months in some study patients. While aggregation made it relatively straightforward to identity and monitor clusters of donor cells, detailed characterization was not possible due to the variable appearance of these grafts. Qualitatively, there was no evidence of an increase in graft size that might indicate tumor formation. On the contrary, overall size was observed to gradually decline over the 12 month period of the study, to the extent that re-dosing is likely necessary for sustained effect in chronic disease.

**TABLE 3:** Optical coherence tomography: study eye.

	0.5 × 10^6^ RPCs	1.0 × 10^6^ RPCs	2.0 × 10^6^ RPCs	3.0 × 10^6^ RPCs	Total [^1^]
	(*N* = 4)	(*N* = 4)	(*N* = 3)	(*N* = 3)	(*N* = 28)
Cohort 1					
**Cystoid macular edema (CME) present**					
Baseline ^[2]^	0/3	0/4	1/3 (33.3%)	0/3	2/27 (7.4%)
Day 28	0/3	0/4	1/3 (33.3%)	0/3	2/27 (7.4%)
Month 3	0/4	0/4	1/3 (33.3%)	0/3	2/28 (7.1%)
Month 6	0/3	0/4	0/3	0/3	1/27 (3.7%)
Month 9	0/4	0/4	0/3	0/3	0/27
Month 12 or early termination	0/4	0/4	0/3	0/3	2/28 (7.1%)
**If CME present, involve foveal center**					
Baseline ^[2]^	0	0	1/1 (100%)	0	2/2 (100%)
Day 28	0	0	1/1 (100%)	0	2/2 (100%)
Month 3	0	0	1/1 (100%)	0	2/2 (100%)
Month 6	0	0	0	0	1/1 (100%)
Month 9	0	0	0	0	0
Month 12 or early termination	0	0	0	0	1/2 (50.0%)
**Cohort 2**					
**Cystoid macular edema (CME) present**					
Baseline ^[2]^	0/4	1/4 (25.0%)	0/3	0/3	2/27 (7.4%)
Day 28	0/4	1/4 (25.0%)	0/3	0/3	2/27 (7.4%)
Month 3	0/4	1/4 (25.0%)	0/3	0/3	2/28 (7.1%)
Month 6	0/4	1/4 (25.0%)	0/3	0/3	1/27 (3.7%)
Month 9	0/3	0/4	0/3	0/3	0/27
Month 12 or early termination	0/4	2/4 (50.0%)	0/3	0/3	2/28 (7.1%)
**If CME present, involve foveal center**					
Baseline ^[2]^	0	1/1 (100%)	0	0	2/2 (100%)
Day 28	0	1/1 (100%)	0	0	2/2 (100%)
Month 3	0	1/1 (100%)	0	0	2/2 (100%)
Month 6	0	1/1 (100%)	0	0	1/1 (100%)
Month 9	0	0	0	0	0
Month 12 or early termination	0	1/2 (50.0%)	0	0	1/2 (50.0%)

^[1]^Total includes both cohorts 1 and 2.

^[2]^Baseline is defined as the value obtained at the baseline visit. If the baseline value is missing, the baseline value will be the last non-missing value recorded prior to the first dose of study drug.

There were no signs of graft rejection despite the absence of systemic immune suppression during the study. Samples from all 28 study subjects were tested for anti-HLA antibodies pre-treatment and at multiple time points through Month 12 of follow-up. Twelve subjects tested positive for anti HLA Class I and/or Class II antibodies, with 11 of these positive at baseline before exposure to jCells. In most instances where there was antibody detected, the antibody specificity was distinct from the HLA type of the donor cells. In a few cases where a subject had broad reactivity to a wide range of HLA antigens at baseline and post-dose, one of the antigens present on the donor cells may have been included in the panel of antigens to which the subjects had antibodies, but there was no pattern in any subject suggestive of an anti-HLA response specific to the injected cells.

One subject was positive for anti-Class I HLA antigen only at Month 12 post-treatment. This subject was in Cohort 1 and received a dose of 2 × 10^6^ RPCs, with stable BCVA in the study eye over the course of the study. The antibody reported was against B15:12, C03:02; the corresponding HLA type of the injected RPCs was B15:13, C04:01G, C08:01G. No other antibodies were detected at the Month 12 or any other time point, and no adverse events were associated with this, making it unlikely that the appearance of this antibody was associated with the treatment 12 months earlier.

The number of subjects reporting treatment-emergent ocular adverse events, and the maximum severity of those adverse events are shown in Table 2. The most commonly reported ocular adverse events were mild conjunctival hemorrhage (10 subjects, 35.7% of all subjects), and mild eye pain (5 subjects, 17.9% of all subjects). These adverse events were as expected given the means of delivery of the RPCs, via intravitreal injection. The low incidence of elevated IOP is expected due to the small volume of injection (50 μL). The incidence of findings consistent with intraocular inflammation (i.e., findings of anterior chamber cell or flare, iritis, iris adhesions, keratic precipitates, or vitreal cells), detected on slit lamp examination of the anterior segment and anterior vitreous, was uncommon and always successfully treated with topical steroids. A total of 7 subjects developed one or more findings associated with intraocular inflammation. Four of the 7 subjects required extension of topical steroid treatment duration beyond normal protocol post-injection drops. Four subjects developed AC cell post-injection, with resolution in 1 subject with routine protocol steroid drop therapy and resolution in the other 3 subjects with extended duration steroid drop therapy. Routine slit lamp exam revealed a number of observations of anterior chamber flare (mild to moderate) in study eyes at one or more time points through month 3 post-treatment, and particularly during the first week post-treatment. While most of these were considered to be not clinically significant, it is suggestive of some mild inflammation that may be associated with the treatment procedure (injection pathway). No anterior chamber flare was noted in any study or non-study eyes beyond 3 months post-treatment.

Optical coherence tomography (OCT) was used to monitor for cystoid macular edema, which is a known manifestation of RP, but might also occur due to treatment-related inflammation (Table 3). Two study subjects had CME present in the study eye at baseline and at several other study time points. One subject had CME reported at Month 12 post-treatment only. There were no other subjects for which CME was noted. There were no reported cases of ERM or other new macular abnormalities, other than the CME in 1 subject noted above.

There was an attempt to determine any therapeutic impact on ERG findings in treated versus untreated eyes during the course of the study; however, the majority of study subjects had ERG results that were significantly abnormal at baseline for both the study eye and the non-study eye, including many reported as “extinguished” or “severely dysfunctional,” which remained unchanged during the course of the study (Tables 4, 5). Thus, it was determined that detailed analysis of ERG results for assessment of efficacy was not warranted and such analyses were not performed. Future studies in patients with similar levels of baseline BCVA impairment will exclude the measurement of ERG.

**TABLE 4:** ERG – study eye, categorical analysis.

	0.5 × 10^6^ RPCs	1.0 × 10^6^ RPCs	2.0 × 10^6^ RPCs	3.0 × 10^6^ RPCs	Total [^1^]
	(*N* = 4)	(*N* = 4)	(*N* = 3)	(*N* = 3)	(*N* = 28)
**Cohort 1**					
**Dark adapted 0.01 ERG (rod response)**					
Baseline ^[2]^	*n* = 4	*n* = 4	*n* = 3	*n* = 3	*n* = 28
Normal	0	0	0	0	0
Abnormal, not clinically significant	0	0	0	0	0
Abnormal, clinically significant	4 (100%)	4 (100%)	3 (100%)	3 (100%)	28 (100%)
Month 6	*n* = 4	*n* = 4	*n* = 3	*n* = 3	*n* = 27
Normal	0	0	0	0	0
Abnormal, not clinically significant	0	1 (25.0%)	0	0	1 (3.7%)
Abnormal, clinically significant	4 (100%)	3 (75.0%)	3 (100%)	3 (100%)	26 (96.3%)
Month 12 or early termination	*n* = 4	*n* = 4	*n* = 3	*n* = 3	*n* = 25
Normal	0	0	0	0	0
Abnormal, not clinically significant	0	1 (25.0%)	0	0	1 (4.0%)
Abnormal, clinically significant	4 (100%)	3 (75.0%)	3 (100%)	3 (100%)	24 (96.0%)
**Dark adapted 3.0 ERG (rod-cone response)**					
Baseline ^[2]^	*n* = 4	*n* = 4	*n* = 3	*n* = 3	*n* = 28
Normal	0	0	0	0	0
Abnormal, not clinically significant	0	0	0	0	0
Abnormal, clinically significant	4 (100%)	4 (100%)	3 (100%)	3 (100%)	28 (100%)
Month 6	*n* = 4	*n* = 4	*n* = 3	*n* = 3	*n* = 27
Normal	0	0	0	0	0
Abnormal, not clinically significant	0	1 (25.0%)	0	0	1 (3.7%)
Abnormal, clinically significant	4 (100%)	3 (75.0%)	3 (100%)	3 (100%)	26 (96.3%)
Month 12 or early termination	*n* = 4	*n* = 4	*n* = 3	*n* = 3	*n* = 25
Normal	0	0	0	0	0
Abnormal, not clinically significant	0	1 (25.0%)	0	0	1 (4.0%)
Abnormal, clinically significant	4 (100%)	3 (75.0%)	3 (100%)	3 (100%)	24 (96.0%)
**Light adapted 3.0 ERG (single flash cone response)**					
Baseline ^[2]^	*n* = 4	*n* = 4	*n* = 3	*n* = 3	*n* = 28
Normal	0	0	0	0	0
Abnormal, not clinically significant	0	0	0	0	0
Abnormal, clinically significant	4 (100%)	4 (100%)	3 (100%)	3 (100%)	28 (100%)
Month 6	*n* = 4	*n* = 4	*n* = 3	*n* = 3	*n* = 27
Normal	0	0	0	0	0
Abnormal, not clinically significant	0	0	0	0	0
Abnormal, clinically significant	4 (100%)	4 (100%)	3 (100%)	3 (100%)	27 (100%)
Month 12 or early termination	*n* = 4	*n* = 4	*n* = 3	*n* = 3	*n* = 25
Normal	0	0	0	0	0
Abnormal, not clinically significant	0	0	0	0	0
Abnormal, clinically significant	4 (100%)	4 (100%)	3 (100%)	3 (100%)	25 (100%)
**Light adapted 3.0 flicker ERG**					
Baseline ^[2]^	*n* = 4	*n* = 4	*n* = 3	*n* = 3	*n* = 28
Normal	0	0	0	0	0
Abnormal, not clinically significant	0	0	0	0	0
Abnormal, clinically significant	4 (100%)	4 (100%)	3 (100%)	3 (100%)	28 (100%)
Month 6	*n* = 4	*n* = 4	*n* = 3	*n* = 2	*n* = 26
Normal	0	0	0	0	0
Abnormal, not clinically significant	0	0	0	0	0
Abnormal, clinically significant	4 (100%)	4 (100%)	3 (100%)	2 (100%)	26 (100%)
Month 12 or early termination	*n* = 4	*n* = 4	*n* = 3	*n* = 3	*n* = 25
Normal	0	0	0	0	0
Abnormal, not clinically significant	0	0	0	0	0
Abnormal, clinically significant	4 (100%)	4 (100%)	3 (100%)	3 (100%)	25 (100%)
**Cohort 2**					
**Dark adapted 0.01 ERG (rod response)**					
Baseline ^[2]^	*n* = 4	*n* = 4	*n* = 3	*n* = 3	*n* = 28
Normal	0	0	0	0	0
Abnormal, not clinically significant	0	0	0	0	0
Abnormal, clinically significant	4 (100%)	4 (100%)	3 (100%)	3 (100%)	28 (100%)
Month 6	*n* = 4	*n* = 4	*n* = 3	*n* = 2	*n* = 27
Normal	0	0	0	0	0
Abnormal, not clinically significant	0	0	0	0	1 (3.7%)
Abnormal, clinically significant	4 (100%)	4 (100%)	3 (100%)	2 (100%)	26 (96.3%)
Month 12 or early termination	*n* = 4	*n* = 1	*n* = 3	*n* = 3	*n* = 25
Normal	0	0	0	0	0
Abnormal, not clinically significant	0	0	0	0	1 (4.0%)
Abnormal, clinically significant	4 (100%)	1 (100%)	3 (100%)	3 (100%)	24 (96.0%)
**Dark adapted 3.0 ERG (rod-cone response)**					
Baseline ^[2]^	*n* = 4	*n* = 4	*n* = 3	*n* = 3	*n* = 28
Normal	0	0	0	0	0
Abnormal, not clinically significant	0	0	0	0	0
Abnormal, clinically significant	4 (100%)	4 (100%)	3 (100%)	3 (100%)	28 (100%)
Month 6	*n* = 4	*n* = 4	*n* = 3	*n* = 2	*n* = 27
Normal	0	0	0	0	0
Abnormal, not clinically significant	0	0	0	0	1 (3.7%)
Abnormal, clinically significant	4 (100%)	4 (100%)	3 (100%)	2 (100%)	26 (96.3%)
Month 12 or early termination	*n* = 4	*n* = 1	*n* = 3	*n* = 3	*n* = 25
Normal	0	0	0	0	0
Abnormal, not clinically significant	0	0	0	0	1 (4.0%)
Abnormal, clinically significant	4 (100%)	1 (100%)	3 (100%)	3 (100%)	24 (96.0%)
**Light adapted 3.0 ERG (single flash cone response)**					
Baseline ^[2]^	*n* = 4	*n* = 4	*n* = 3	*n* = 3	*n* = 28
Normal	0	0	0	0	0
Abnormal, not clinically significant	0	0	0	0	0
Abnormal, clinically significant	4 (100%)	4 (100%)	3 (100%)	3 (100%)	28 (100%)
Month 6	*n* = 4	*n* = 4	*n* = 3	*n* = 2	*n* = 27
Normal	0	0	0	0	0
Abnormal, not clinically significant	0	0	0	0	0
Abnormal, clinically significant	4 (100%)	4 (100%)	3 (100%)	2 (100%)	27 (100%)
Month 12 or early termination	*n* = 4	*n* = 1	*n* = 3	*n* = 3	*n* = 25
Normal	0	0	0	0	0
Abnormal, not clinically significant	0	0	0	0	0
Abnormal, clinically significant	4 (100%)	1 (100%)	3 (100%)	3 (100%)	25 (100%)
**Light adapted 3.0 flicker ERG**					
Baseline ^[2]^	*n* = 4	*n* = 4	*n* = 3	*n* = 3	*n* = 28
Normal	0	0	0	0	0
Abnormal, not clinically significant	0	0	0	0	0
Abnormal, clinically significant	4 (100%)	4 (100%)	3 (100%)	3 (100%)	28 (100%)
Month 6	*n* = 4	*n* = 4	*n* = 3	*n* = 2	*n* = 26
Normal	0	0	0	0	0
Abnormal, not clinically significant	0	0	0	0	0
Abnormal, clinically significant	4 (100%)	4 (100%)	3 (100%)	2 (100%)	26 (100%)
Month 12 or early termination	*n* = 4	*n* = 1	*n* = 3	*n* = 3	*n* = 25
Normal	0	0	0	0	0
Abnormal, not clinically significant	0	0	0	0	0
Abnormal, clinically significant	4 (100%)	1 (100%)	3 (100%)	3 (100%)	25 (100%)

^[1]^Total includes both cohorts 1 and 2.

^[2]^Baseline is defined as the value obtained at the baseline visit. If the baseline value is missing, the baseline value will be the last non-missing value recorded prior to the first dose of study drug.

**TABLE 5:** ERG – non-study eye, categorical analysis.

	0.5 × 10^6^ RPCs	1.0 × 10^6^ RPCs	2.0 × 10^6^ RPCs	3.0 × 10^6^ RPCs	Total [^1^]
	(*N* = 4)	(*N* = 4)	(*N* = 3)	(*N* = 3)	(*N* = 28)
**Cohort 1**					
**Dark adapted 0.01 ERG (rod response)**					
Baseline ^[2]^	*n* = 4	*n* = 4	*n* = 3	*n* = 3	*n* = 28
Normal	0	0	0	0	0
Abnormal, not clinically significant	0	0	0	0	0
Abnormal, clinically significant	4 (100%)	4 (100%)	3 (100%)	3 (100%)	28 (100%)
Month 6	*n* = 4	*n* = 4	*n* = 3	*n* = 3	*n* = 27
Normal	0	0	0	0	0
Abnormal, not clinically significant	0	0	0	0	0
Abnormal, clinically significant	4 (100%)	4 (100%)	3 (100%)	3 (100%)	27 (100%)
Month 12 or early termination	*n* = 4	*n* = 4	*n* = 3	*n* = 3	*n* = 25
Normal	0	0	0	0	0
Abnormal, not clinically significant	0	0	0	0	0
Abnormal, clinically significant	4 (100%)	4 (100%)	3 (100%)	3 (100%)	25 (100%)
**Dark adapted 3.0 ERG (rod-cone response)**					
Baseline ^[2]^	*n* = 4	*n* = 4	*n* = 3	*n* = 3	*n* = 28
Normal	0	0	0	0	0
Abnormal, not clinically significant	0	0	0	0	0
Abnormal, clinically significant	4 (100%)	4 (100%)	3 (100%)	3 (100%)	28 (100%)
Month 6	*n* = 4	*n* = 4	*n* = 3	*n* = 3	*n* = 27
Normal	0	0	0	0	0
Abnormal, not clinically significant	0	0	0	0	0
Abnormal, clinically significant	4 (100%)	4 (100%)	3 (100%)	3 (100%)	27 (100%)
Month 12 or early termination	*n* = 4	*n* = 4	*n* = 3	*n* = 3	*n* = 25
Normal	0	0	0	0	0
Abnormal, not clinically significant	0	0	0	0	0
Abnormal, clinically significant	4 (100%)	4 (100%)	3 (100%)	3 (100%)	25 (100%)
**Light adapted 3.0 ERG (single flash cone response)**					
Baseline ^[2]^	*n* = 4	*n* = 4	*n* = 3	*n* = 3	*n* = 28
Normal	0	0	0	0	0
Abnormal, not clinically significant	0	0	0	0	0
Abnormal, clinically significant	4 (100%)	4 (100%)	3 (100%)	3 (100%)	28 (100%)
Month 6	*n* = 4	*n* = 4	*n* = 3	*n* = 3	*n* = 27
Normal	0	0	0	0	0
Abnormal, not clinically significant	0	0	0	0	0
Abnormal, clinically significant	4 (100%)	4 (100%)	3 (100%)	3 (100%)	27 (100%)
Month 12 or early termination	*n* = 4	*n* = 4	*n* = 3	*n* = 3	*n* = 25
Normal	0	0	0	0	0
Abnormal, not clinically significant	0	0	0	0	0
Abnormal, clinically significant	4 (100%)	4 (100%)	3 (100%)	3 (100%)	25 (100%)
**Light adapted 3.0 Flicker ERG**					
Baseline ^[2]^	*n* = 4	*n* = 4	*n* = 3	*n* = 3	*n* = 28
Normal	0	0	0	0	0
Abnormal, not clinically significant	0	0	0	0	0
Abnormal, clinically significant	4 (100%)	4 (100%)	3 (100%)	3 (100%)	28 (100%)
Month 6	*n* = 4	*n* = 4	*n* = 3	*n* = 2	*n* = 26
Normal	0	0	0	0	0
Abnormal, not clinically significant	0	0	0	0	0
Abnormal, clinically significant	4 (100%)	4 (100%)	3 (100%)	2 (100%)	26 (100%)
Month 12 or early termination	*n* = 4	*n* = 4	*n* = 3	*n* = 3	*n* = 25
Normal	0	0	0	0	0
Abnormal, not clinically significant	0	0	0	0	0
Abnormal, clinically significant	4 (100%)	4 (100%)	3 (100%)	3 (100%)	25 (100%)
**Cohort 2**					
**Dark adapted 0.01 ERG (rod response)**					
Baseline ^[2]^	*n* = 4	*n* = 4	*n* = 3	*n* = 3	*n* = 28
Normal	0	0	0	0	0
Abnormal, not clinically significant	0	0	0	0	0
Abnormal, clinically significant	4 (100%)	4 (100%)	3 (100%)	3 (100%)	28 (100%)
Month 6	*n* = 4	*n* = 4	*n* = 3	*n* = 2	*n* = 27
Normal	0	0	0	0	0
Abnormal, not clinically significant	0	0	0	0	0
Abnormal, clinically significant	4 (100%)	4 (100%)	3 (100%)	2 (100%)	27 (100%)
Month 12 or early termination	*n* = 4	*n* = 1	*n* = 3	*n* = 3	*n* = 25
Normal	0	0	0	0	0
Abnormal, not clinically significant	0	0	0	0	0
Abnormal, clinically significant	4 (100%)	1 (100%)	3 (100%)	3 (100%)	25 (100%)
**Dark adapted 3.0 ERG (rod-cone response)**					
Baseline ^[2]^	*n* = 4	*n* = 4	*n* = 3	*n* = 3	*n* = 28
Normal	0	0	0	0	0
Abnormal, not clinically significant	0	0	0	0	0
Abnormal, clinically significant	4 (100%)	4 (100%)	3 (100%)	3 (100%)	28 (100%)
Month 6	*n* = 4	*n* = 4	*n* = 3	*n* = 2	*n* = 27
Normal	0	0	0	0	0
Abnormal, not clinically significant	0	0	0	0	0
Abnormal, clinically significant	4 (100%)	4 (100%)	3 (100%)	2 (100%)	27 (100%)
Month 12 or early termination	*n* = 4	*n* = 1	*n* = 3	*n* = 3	*n* = 25
Normal	0	0	0	0	0
Abnormal, not clinically significant	0	0	0	0	0
Abnormal, clinically significant	4 (100%)	1 (100%)	3 (100%)	3 (100%)	25 (100%)
**Light adapted 3.0 ERG (single flash cone response)**					
Baseline ^[2]^	*n* = 4	*n* = 4	*n* = 3	*n* = 3	*n* = 28
Normal	0	0	0	0	0
Abnormal, not clinically significant	0	0	0	0	0
Abnormal, clinically significant	4 (100%)	4 (100%)	3 (100%)	3 (100%)	28 (100%)
Month 6	*n* = 4	*n* = 4	*n* = 3	*n* = 2	*n* = 27
Normal	0	0	0	0	0
Abnormal, not clinically significant	0	0	0	0	0
Abnormal, clinically significant	4 (100%)	4 (100%)	3 (100%)	3 (100%)	28 (100%)
Month 12 or early termination	*n* = 4	*n* = 1	*n* = 3	*n* = 3	*n* = 25
Normal	0	0	0	0	0
Abnormal, not clinically significant	0	0	0	0	0
Abnormal, clinically significant	4 (100%)	1 (100%)	3 (100%)	3 (100%)	25 (100%)
**Light adapted 3.0 flicker ERG**					
Baseline ^[2]^	*n* = 4	*n* = 4	*n* = 3	*n* = 3	*n* = 28
Normal	0	0	0	0	0
Abnormal, not clinically significant	0	0	0	0	0
Abnormal, clinically significant	4 (100%)	4 (100%)	3 (100%)	3 (100%)	28 (100%)
Month 6	*n* = 4	*n* = 4	*n* = 3	*n* = 2	*n* = 26
Normal	0	0	0	0	0
Abnormal, not clinically significant	0	0	0	0	0
Abnormal, clinically significant	4 (100%)	4 (100%)	3 (100%)	2 (100%)	26 (100%)
Month 12 or early termination	*n* = 4	*n* = 1	*n* = 3	*n* = 3	*n* = 25
Normal	0	0	0	0	0
Abnormal, not clinically significant	0	0	0	0	0
Abnormal, clinically significant	4 (100%)	1 (100%)	3 (100%)	3 (100%)	25 (100%)

^[1]^Total includes both cohorts 1 and 2.

^[2]^Baseline is defined as the value obtained at the baseline visit. If the baseline value is missing, the baseline value will be the last non-missing value recorded prior to the first dose of study drug.

Slit lamp and fundus examination were scheduled at each study visit except on the day of treatment. In general, there were only a few eye structures or findings for which differences/changes were noted only in study eyes. These include reports in 2 subjects of clinically significant conjunctival findings at Day 2 post-treatment. No other assessments of conjunctiva were considered clinically significant. These two reports and most other instances of abnormal findings were associated with subconjunctival hemorrhages and were reported as mild adverse events related to study treatment.

In general, the observations of the cornea were similar between study eyes and non-study eyes, with a single exception. One subject with a normal cornea at baseline was reported subsequently as abnormal, clinically significant by Day 7 post-treatment. This was associated with trace keratic precipitates and was reported as a mild adverse event. The observations of this subject’s cornea varied between normal and abnormal, clinically significant through Month 3 post-treatment, after which all observations were reported as normal.

Fluorescein angiography was performed on most subjects at baseline and at Month 12 post-treatment. No observations of active leakage or ischemia were reported for any study eye at any time point. “Other” observations reported from these assessments were generally descriptions that were consistent with retinitis pigmentosa, such as vascular attenuation, window defects associated with RPE atrophy, and blockage associated with bone spicules.

Fundus autofluorescence assessments of the study eye generally showed stable findings between the baseline visit and the 6- and 12-month post-treatment visits, with 71% of study subjects (*n* = 20) demonstrating clinically significant abnormal results at all three time points. Only one subject was reported to have normal results at baseline, progressing to abnormal, not clinically significant findings at the 6-month post-treatment visit.

IOP was generally stable over the course of the study and comparable between the study and non-study eyes with one exception. One subject had elevated IOP (42 mmHg) in the study eye at Day 1 post-treatment, which resolved the following day following medication. There were no other adverse events reported relating to increased IOP.

The efficacy analyses were considered exploratory. Baseline scores for BCVA (E-ETDRS letters correct) for Cohort 1 study eyes (range 0–35 letters correct at baseline) were considerably lower than for Cohort 2 (range 36–60 letters correct at baseline), with no overlap between cohorts; although there were large intra-cohort ranges in BCVA score. Positive changes from baseline were reported in all treated eye groups, with the suggestion of a pronounced dose-response at highest RPC dose levels (Table 6). Overall, there was no discernible BCVA change in untreated eyes.

**TABLE 6:** Mean E-ETDRS BCVA change from baseline for treated and untreated eyes by cohort and retinal progenitor cell (RPC) dose level.

	Baseline (BL)	3 months	6 months	9 months	12 months	
RPC dose (Cells)	Mean letter score (SD)	Mean letter change from baseline (SD)	Mean letter change from baseline (SD)	Mean letter change from baseline (SD)	Mean letter score (SD)	Mean letter change from baseline (SD)
**Cohort 1 (treated eye)**						
0.5 × 10^6^ *n* = 4	15.5 (18.19)	2.5 (5.00)	2.0 (3.37)	3.3 (5.25)	18.8 (21.09)	3.3 (5.19)
1.0 × 10^6^ *n* = 4	10.5 (13.08)	2.8 (1.71)	1.3 (3.30)	6.3 (5.32)	14.3 (12.50)	3.8 (5.85)
2.0 × 10^6^ *n* = 3	11.3 (17.93)	2.3 (4.93)	1.7 (2.89)	−0.7 (1.15)	13.0 (20.81)	1.7 (2.89)
3.0 × 10^6^ *n* = 3	8.7 (15.01)	5.3 (5.51)	7.0 (7.55)	4.7 (4.51)	16.7 (19.43)	8.0 (6.93)
**Cohort 1 (untreated eye)**						
0.5 × 10^6^ *n* = 4	21.3 (24.57)	−2.0 (5.35)	−3.5 (7.42)	−2.3 (4.57)	20.0 (22.70)	−1.3 (4.57)
1.0 × 10^6^ *n* = 4	21.0 (28.44)	0.5 (4.04)	2.3 (2.22)	4.3 (6.40)	23.5 (30.97)	2.5 (3.00)
2.0 × 10^6^ *n* = 3	24.7 (27.59)	0.7 (3.06)	0.0 (6.24)	−6.7 (14.57)	21.7 (23.12)	−3.0 (7.55)
3.0 × 10^6^ *n* = 3	40.0 (13.00)	3.3 (0.58)	3.0 (2.65)	3.0 (4.36)	42.0 (11.27)	2.0 (1.73)
**Cohort 1 (difference between treated and untreated eyes)**						
0.5 × 10^6^ *n* = 4	−5.8 (8.02)	4.5 (10.34)	5.5 (10.54)	5.5 (9.81)	−1.3 (3.95)	4.50 (9.68)
1.0 × 10^6^ *n* = 4	−10.5 (17.99)	2.3 (3.20)	−1.0 (4.69)	2.0 (4.97)	−9.3 (24.72)	1.3 (7.14)
2.0 × 10^6^ *n* = 3	−13.3 (11.02)	1.7 (7.37)	1.7 (8.50)	6.0 (14.93)	−8.7 (10.50)	4.7 (10.02)
3.0 × 10^6^ *n* = 3	−31.3 (24.01)	2.0 (5.00)	4.0 (8.72)	1.7 (8.74)	−25.3 (28.57)	6.0 (5.20)
**Cohort 2 (treated eye)**						
0.5 × 10^6^ *n* = 4	44.5 (10.88)	3.8 (2.06)	4.5 (7.33)	4.8 (4.03)	46.5 (13.30)	2.0 (4.16)
1.0 × 10^6^ *n* = 4	54.3 (6.95)	0.8 (5.38)	5.0 (4.32)	3.5 (5.20)	56.8 (8.62)	2.5 (4.80)
2.0 × 10^6^ *n* = 3	44.3 (9.71)	2.0 (10.58)	4.3 (6.03)	6.7 (2.08)	48.7 (13.20)	4.3 (3.51)
3.0 × 10^6^ *n* = 3	50.3 (11.55)	3.3 (2.08)	6.0 (5.29)	4.3 (2.52)	55.0 (5.20)	4.7 (6.35)
**Cohort 2 (untreated eye)**						
0.5 × 10^6^ *n* = 4	57.8 (4.19)	3.5 (4.65)	4.5 (3.79)	4.3 (1.26)	61.5 (7.42)	3.8 (3.50)
1.0 × 10^6^ *n* = 4	63.5 (2.52)	2.3 (3.10)	2.8 (5.44)	−0.5 (2.65)	65.3 (6.40)	1.8 (4.19)
2.0 × 10^6^ *n* = 3	49.7 (14.22)	−0.7 (7.23)	−6.7 (10.02)	0.7 (11.50)	49.0 (23.81)	−0.7 (16.56)
3.0 × 10^6^ *n* = 3	67.7 (10.50)	−5.0 (1.73)	−0.7 (1.53)	−5.0 (1.00)	60.3 (14.57)	−7.3 (5.13)
**Cohort 2 (difference between treated and untreated eyes)**						
0.5 × 10^6^ *n* = 4	−13.3 (6.90)	0.3 (2.87)	0.0 (7.16)	0.5 (3.70)	−15.0 (6.48)	−1.8 (2.99)
1.0 × 10^6^ *n* = 4	−9.3 (7.98)	−1.5 (7.00)	2.3 (3.30)	4.0 (3.74)	−8.5 (12.58)	0.8 (6.50)
2.0 × 10^6^ *n* = 3	−5.3 (5.13)	2.7 (4.04)	11.0 (11.14)	6.0 (11.14)	−0.3 (13.87)	5.0 (15.10)
3.0 × 10^6^ *n* = 3	−17.3 (5.51)	8.3 (3.79)	6.7 (6.66)	9.3 (3.51)	−5.3 (9.61)	12.0 (11.36)
**All subjects (treated eye)**						
0.5 × 10^6^ *n* = 8	30.0 (20.81)	3.1 (3.60)	3.3 (5.44)	4.0 (4.41)	32.6 (22.06)	2.6 (4.41)
1.0 × 10^6^ *n* = 8	32.4 (25.31)	1.8 (3.85)	3.1 (4.09)	4.9 (5.08)	35.5 (24.8)	3.1 (5.00)
2.0 × 10^6^ *n* = 6	27.8 (22.20)	2.2 (7.39)	3.0 (4.47)	3.0 (4.29)	30.8 (24.99)	3.0 (3.22)
3.0 × 10^6^ *n* = 6	29.5 (25.77)	4.3 (3.88)	6.5 (5.86)	4.5 (3.27)	35.8 (24.55)	6.3 (6.22)
**All subjects (untreated eye)**						
0.5 × 10^6^ *n* = 8	39.5 (25.43)	0.8 (5.50)	0.5 (6.93)	1.0 (4.66)	40.8 (27.14)	1.3 (4.62)
1.0 × 10^6^ *n* = 8	42.3 (29.42)	1.4 (3.46)	2.5 (3.85)	1.9 (5.19)	44.4 (30.44)	2.1 (3.40)
2.0 × 10^6^ *n* = 6	37.2 (23.94)	0.0 (5.02)	−3.3 (8.31)	−3.0 (12.41)	35.3 (25.78)	−1.8 (11.58)
3.0 × 10^6^ *n* = 6	53.8 (18.48)	−0.8 (4.71)	1.2 (2.79)	−1.0 (5.22)	51.2 (15.38)	−2.7 (6.15)
**All subjects (difference between treated and untreated eyes)**						
0.5 × 10^6^ *n* = 8	−9.5 (8.00)	2.4 (7.39)	2.8 (8.84)	3.0 (7.37)	−8.1 (8.87)	1.4 (7.42)
1.0 × 10^6^ *n* = 8	−9.9 (12.88)	0.4 (5.42)	0.6 (4.14)	3.0 (4.21)	−8.9 (18.16)	1.0 (6.32)
2.0 × 10^6^ *n* = 6	−9.3 (8.85)	2.2 (5.34)	6.3 (10.23)	6.0 (11.78)	−4.5 (11.91)	4.8 (11.46)
3.0 × 10^6^ *n* = 6	−24.3 (17.36)	5.2 (5.27)	5.3 (7.09)	5.5 (7.29)	−15.3 (21.99)	9.0 (8.56)

Untreated fellow eyes demonstrated a range of mean changes from baseline at the 3-month examination intervals, which included deterioration at each of these intervals as evidenced by a negative value at lower end of range. Only one time point (9 months) for treated eyes showed changes from baseline that included a negative value. The range of mean change from baseline for treated eyes included improvements of more than 5 letters (except Cohort 2 at Month 3 timepoint), whereas none of the untreated eyes showed similar changes. Individual subjects’ BCVA results (E-ETDRS Letters Correct) for treated and untreated fellow eyes across the 12-month study period are shown in Supplementary Table 1.

**FIGURE 2:**
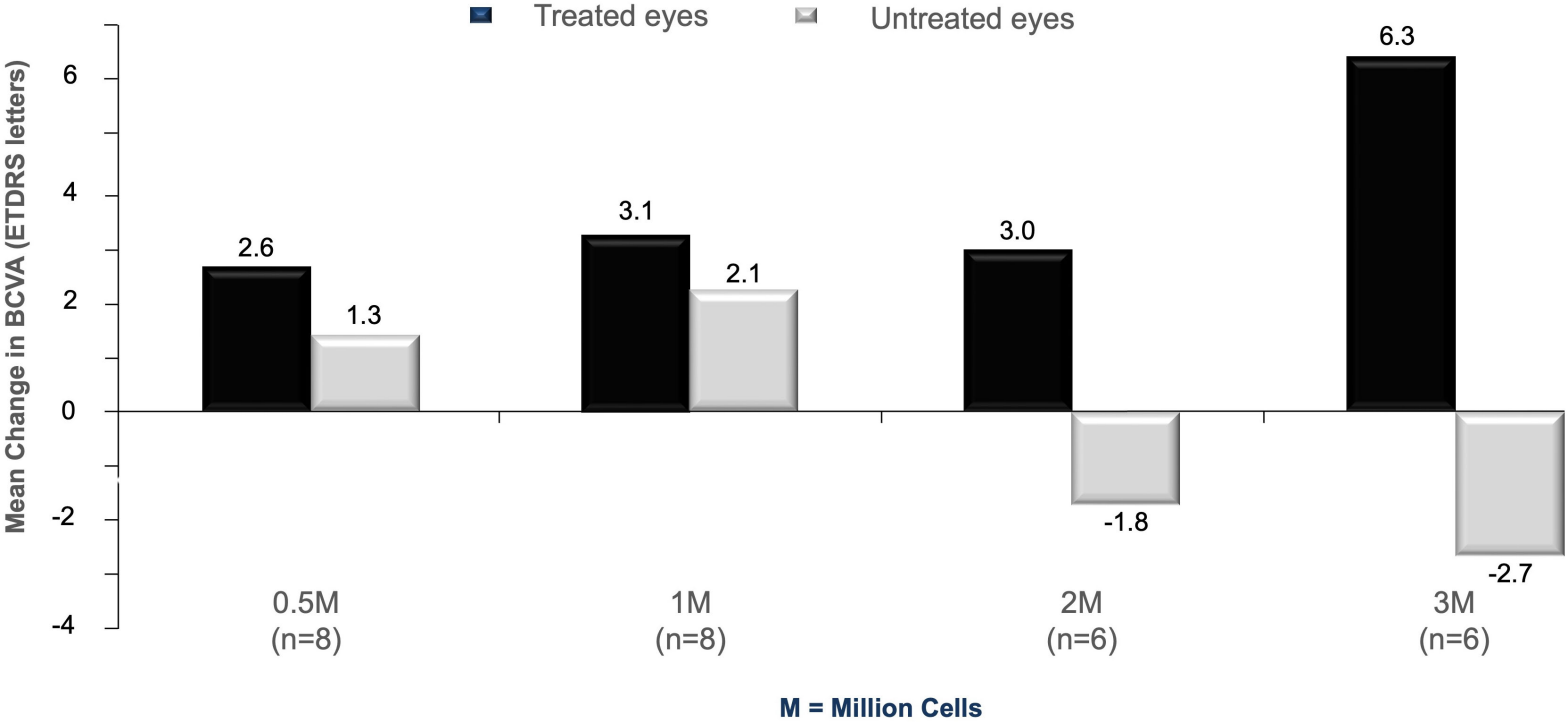
Mean change in BCVA from baseline in study subjects at 12 months post-treatment. Data from cell-treated eyes (black histograms) are compared to untreated fellow eyes (gray histograms) across the four different doses used in the study (0.5, 1, 2, and 3 million cells).

Mean change in BCVA from pre-treatment to Month 12 in the treated eye compared with the untreated eye (mean change in BCVA in treated eye over 12 months minus change in untreated eye over 12 months) was 1.4 letters for the 0.5 × 10^6^ RPC group, 1.0 letters for the 1.0 × 10^6^ group, 4.8 letters for the 2.0 × 10^6^ group, and 9.0 letters for the 3.0 × 10^6^ group (Figure 2 and Table 6). The difference in mean change in BCVA scores (treated vs untreated fellow eyes) is suggestive of a more pronounced dose-response at the highest dose levels.

Mean change in BCVA over 12 months (treated vs untreated fellow eyes) suggests potential improvements in treated eyes when the 8 patients without measurable BCVA at baseline were excluded. ETDRS letter scores corresponding to Snellen acuities of <20/800 (i.e., ≤ 2 ETDRS letters) are considered unmeasurable. Differences at 12 months were 1.8 letters for the 0.5 × 10^6^ dose group, 0.2 letters for the 1.0 × 10^6^ group, 7.5 letters for the 2.0 × 10^6^ group, and 11.3 letters for 3.0 × 10^6^ dose group, further supporting the potential dose-response effect of RPCs observed at the highest dose levels.

These trends were supported by the categorical analysis of BCVA data, which also suggest improvements in treated eyes compared with untreated eyes, particularly at the highest dose levels (Table 7).

**TABLE 7:** BCVA change post-treatment in eyes treated with 3.0 × 10^6^ RPC (cohort 1 and cohort 2): number of letters.

Time post-treatment	3.0 × 10^6^ RPC	
	Treated eye (*N* = 6)	Non-treated eye (*N* = 6)
**Month 3**		
≥5 Letters increase	3 (50.0%)	0
≥10 Letters increase	1 (16.7%)	0
≥15 Letters increase	0	0
**Month 6**		
≥5 Letters increase	3 (50.0%)	1 (16.7%)
≥10 Letters increase	2 (33.3%)	0
≥15 Letters increase	1 (16.7%)	0
**Month 9**		
≥5 Letters increase	3 (50.0%)	1 (16.7%)
≥10 Letters increase	0	0
≥15 Letters increase	0	0
**Month 12**		
≥5 Letters increase	3 (50.0%)	0
≥10 Letters increase	3 (50.0%)	0
≥15 Letters increase	0	0

During the study, the Sponsor became aware that the methods being used to assess visual field varied between sites and were not particularly sensitive for the low vision patient population being studied. Therefore, it was determined that visual field results would be summarized by proportion of subjects with clinically significant abnormalities at each time point (baseline, 6 months and end of study). In total, 76.9% of study subjects had clinically significant abnormalities in the visual field examination of the study eye at baseline,

88.5% at month 6 after treatment and 95.2% at month 12 after treatment.

Proportions of clinically significant results were similar for the non-study eye at each timepoint. In general, there were few meaningful changes reported for this assessment during the course of the study.

During the study, informal feedback from the clinical staff suggested that subjects were reporting visual changes that may not have been captured by the then-current assessments, especially in the low vision cohort. Therefore, a protocol amendment added the option to explore the use of further visual assessment instruments and questionnaires. A low vision expert was added to the team to visit study sites to explore the viability of use of further assessment methods in this study population for potential use in future studies. The additional exploratory endpoints were not conducted in all subjects and any data generated were not stored.

### 3.1 Patient-reported outcomes

Informal feedback from investigational staff reported subtle visual changes not captured by standard clinical assessments, including in patients who did not necessarily show measurable improvements in visual acuity. Patient-reported vision changes were subsequently captured retrospectively (from visit notes) and prospectively. Patients commonly reported increased sensitivity to light, but there were also reports of improved object recognition, color discrimination, and reading ability; even in patients without any measured improvement in BCVA.

## 4 Discussion

Primarily an evaluation of intravitreal RPC safety, treatment was generally well-tolerated and associated with minimal discomfort. Small reticular or dispersed opaque globules (consistent with aggregated donor cells) were observed in the vitreous cavity at various time points, but with no indications of an increased donor cell burden, cell proliferation, or tumor formation. There were no signs of RPC graft rejection, despite the absence of immune suppression therapies.

The jCell intravitreal injection of up to 3.0 × 10^6^ RPCs appeared safe and associated with relatively few transient and mostly mild to moderate events in patients. There were some cases of intraocular inflammation, however, the inflammation always responded well to observation or topical steroids. There was one case of CME reported at Month 12 post-treatment; no cases of ERM or other new macular abnormalities were reported.

Although each patient received a single dose of RPCs in the eye with the poorest vision, considered prudent for this initial formal clinical study, the eventual goal is bilateral treatment. Future key long term safety goals must include monitoring for any potential risk of tumor formation, immune rejection, treatment-associated disease acceleration, persistent increases in IOP, clinically significant epiretinal membrane, retinal detachment, neovascularization, or endophthalmitis. None of these events were observed in this study.

This prospective study in patients with a progressive neurodegenerative retinal disease provides encouraging exploratory efficacy results suggestive of an improvement in visual function. Measured changes in BCVA between treated and untreated eyes were nominally positive at all dose levels, with the suggestion of a possible dose-response at the highest dose levels. When patients without measurable BCVA at baseline were excluded from the analysis (i.e., those with end-stage disease) the mean change in BCVA from baseline in the treated eye appeared to be greater. While this study suggests there may be a beneficial effect on visual acuity, the data need to be interpreted with caution since this was a small study which was not designed or powered to rigorously assess efficacy. However, the observations are consistent with the hypothesis that RPCs may have greater potential to benefit visual function in patients prior to end-stage disease, and therefore future clinical studies will be conducted in patients with baseline BCVA of at least 20/800 Snellen in the treated eye. Moreover, other potential visual changes including increased sensitivity to light, improved object recognition, color discrimination, and reading ability appeared to occur in some patients without a measurable BCVA improvement. Such patient-reported outcomes are motivation to search for additional relevant endpoints in this population.

The mean BCVA data presented are associated with large standard deviations, indicating the wide variability among a small study population in each cohort. Similar differences were noted in the mean scores of the untreated eyes for each cohort and dose level. These large standard deviations pose important challenges in interpreting the data and for future study design. This study was designed primarily to assess safety of a range of doses, and to gain insights into potential dose response in two different patient cohorts, defined by baseline BCVA. As such each subject subgroup, defined by a combination of dose and baseline BCVA, consists of 3–4 subjects. It is therefore not possible in this study to rigorously assess the likely impact of other baseline characteristics, independent of the dose used. The greater apparent response in subjects with ≥20/800 baseline Snellen acuities, compared to those with Snellen acuities of <20/800 (i.e., ≤ 2 ETDRS letters), suggests that the degree of disease progression may influence outcomes in subjects treated with RPCs. However, whether there are particular anatomical markers, measures of residual retinal function or genetic subtypes which are predictive of response will need to be evaluated in future studies.

As may be expected from treating the eye with poorest vision for each patient, mean BCVA scores for non-study eyes were slightly higher overall.

At 12 months, the study data are suggestive of improved visual acuity, with the largest difference (9 letters between test and control) observed in the 3.0 × 10^6^ (highest) dose group, and in patients without end-stage BCVA impairment (11.3 letters between test and control). Whether sex, gender or age affect outcomes was not analysed due to the small sample size in this open label safety study. This limitation will be addressed in future larger controlled studies. The study outcomes have been discussed with the FDA and provide the basis for the Phase IIb study design.

The only FDA-approved treatment for RP, voretigene neparvovec, is approved for use in patients with biallelic RPE65 mutations. RPE65 mutations account for approximately 2% of recessive RP (Morimura et al., 1998). Furthermore, many current development programs, including other gene therapies, also target specific RP genotypes, whereas RPCs offer a novel gene-agnostic approach with the potential for use in the broad RP patient population. The RPC technology of jCell has received RMAT (Regenerative Medicine Advanced Therapy) designation from the FDA. This is a special regulatory pathway established to accelerate the approval process for innovative regenerative medicine therapies. A regenerative medicine may receive RMAT designation if FDA believes there is early clinical evidence indicating the potential to address unmet medical needs or offer significant improvements over existing treatments for serious or life-threatening diseases or conditions.

## 5 Conclusion

In this prospective, multicenter Phase I/IIa safety study in adult patients with RP, a single intravitreal injection of jCell was well-tolerated with minimal discomfort and no indication of graft rejection, despite the allogeneic context and lack of systemic immune suppression.

While outcomes from this small safety study are preliminary, the data are suggestive of an improvement of visual acuity in some patients with this progressive neurodegenerative retinal disease. Apparent improvements were most marked in patients without end-stage disease and in the highest dose group. Together, these data support further clinical development of this treatment approach.

This study has several limitations. It was a small, early-phase trial designed primarily to evaluate safety and tolerability and was not powered to demonstrate efficacy. While exploratory analyses suggested a possible functional signal and an apparent dose–response effect, these findings should be interpreted cautiously given the limited sample size, open-label design, and variability in baseline disease severity. Treatment was administered to the eye with the poorest vision, which may have reduced the likelihood of detecting functional improvement in patients with advanced disease. In addition, the range of doses tested was limited (up to 3.0 × 10^6^ RPCs); given the suggestion of greater benefit at higher doses, future studies should explore higher dose levels in larger, randomized, controlled cohorts to confirm safety and rigorously assess efficacy.

## Data Availability

The original contributions presented in this study are included in this article/Supplementary material, further inquiries can be directed to the corresponding author.
