# Supplementary material for: Retinal progenitor cells (jCell) for retinitis pigmentosa
Source: Front Cell Neurosci. 2025 Aug 25;19:1646156. doi: 10.3389/fncel.2025.1646156 (PMC12414927; doi:10.3389/fncel.2025.1646156)
Supplement: Supplementary file 1 [file Table_1.docx]

**Supplementary Material**

1. **Supplementary Table**

**Supplementary Table A: BCVA (E-ETDRS Letters Correct) for Treated and Untreated Fellow Eyes for All Subjects Across 12-Month Study Period**

|  | | | | **Study Eye**  **(Letters Correct)** | | | | | | |
| --- | --- | --- | --- | --- | --- | --- | --- | --- | --- | --- |
| **Subject** | **jCell Dose Group** | **Cohort** | **Age** | **Baseline** | **Day 28** | **Month 3** | **Month 6** | **Month 9** | **Month 12** | **Change from Baseline to Month 12** |
| 1 | 0.5M | 1 | 64 | 27 | 38 | 37 | 34 | 38 | 38 | 11.0 |
| 2 | 0.5M | 1 | 51 | 35 | 36 | 35 | 35 | 37 | 36 | 1.0 |
| 3 | 0.5M | 1 | 58 | 0 | 1 | 0 | 1 | 0 | 1 | 1.0 |
| 4 | 0.5M | 1 | 28 | 0 | 0 | 0 | 0 | 0 | 0 | 0.0 |
| 5 | 0.5M | 2 | 69 | 44 | 47 | 48 | 59 | 51 | 51 | 7.0 |
| 6 | 0.5M | 2 | 38 | 60 | 66 | 66 | 62 | 59 | 63 | 3.0 |
| 7 | 0.5M | 2 | 57 | 38 | 42 | 39 | 41 | 43 | 39 | 1.0 |
| 8 | 0.5M | 2 | 18 | 36 | 46 | 40 | 34 | 44 | 33 | -3.0 |
| 9 | 1.0M | 1 | 36 | 0 | 0 | 2 | 2 | 5 | 5 | 5.0 |
| 10 | 1.0M | 1 | 59 | 0 | 1 | 1 | 1 | 2 | 2 | 2.0 |
| 11 | 1.0M | 1 | 45 | 15 | 23 | 20 | 20 | 29 | 26 | 11.0 |
| 12 | 1.0M | 1 | 40 | 27 | 25 | 30 | 24 | 31 | 24 | -3.0 |
| 13 | 1.0M | 2 | 52 | 60 | 62 | 60 | 61 | 62 | 60 | 0.0 |
| 14 | 1.0M | 2 | 30 | 60 | 66 | 60 | 65 | 62 | 63 | 3.0 |
| 15 | 1.0M | 2 | 60 | 51 | 61 | 59 | 62 | 62 | 60 | 9.0 |
| 16 | 1.0M | 2 | 56 | 46 | 49 | 41 | 49 | 45 | 44 | -2.0 |
| 17 | 2.0M | 1 | 72 | 0 | 0 | 0 | 0 | 0 | 0 | 0.0 |
| 18 | 2.0M | 1 | 29 | 2 | 1 | 1 | 2 | 0 | 2 | 0.0 |
| 19 | 2.0M | 1 | 62 | 32 | 30 | 40 | 37 | 32 | 37 | 5.0 |
| 20 | 2.0M | 2 | 42 | 36 | 7 | 26 | 41 | 41 | 37 | 1.0 |
| 21 | 2.0M | 2 | 73 | 42 | 50 | 52 | 40 | 48 | 46 | 4.0 |
| 22 | 2.0M | 2 | 54 | 55 | 61 | 61 | 65 | 64 | 63 | 8.0 |
| 23 | 3.0M | 1 | 48 | 0 | 0 | 0 | 0 | 0 | 0 | 0.0 |
| 24 | 3.0M | 1 | 54 | 0 | 16 | 11 | 15 | 9 | 12 | 12.0 |
| 25 | 3.0M | 1 | 67 | 26 | 30 | 31 | 32 | 31 | 38 | 12.0 |
| 26 | 3.0M | 2 | 48 | 57 | 59 | 58 | 59 | 61 | 58 | 1.0 |
| 27 | 3.0M | 2 | 28 | 57 | 59 | 62 | 61 | 59 | 58 | 1.0 |
| 28 | 3.0M | 2 | 40 | 37 | 44 | 41 | 49 | 44 | 49 | 12.0 |

|  | | | | **Fellow Eye**  **(Letters Correct)** | | | | | | |
| --- | --- | --- | --- | --- | --- | --- | --- | --- | --- | --- |
| **Subject** | **jCell Dose Group** | **Cohort** | **Age** | **Baseline** | **Day 28** | **Month 3** | **Month 6** | **Month 9** | **Month 12** | **Change from Baseline to Month 12** |
| 1 | 0.5M | 1 | 64 | 44 | 43 | 34 | 30 | 35 | 36 | -8.0 |
| 2 | 0.5M | 1 | 51 | 41 | 38 | 42 | 38 | 40 | 43 | 2.0 |
| 3 | 0.5M | 1 | 58 | 0 | 1 | 1 | 3 | 1 | 1 | 1.0 |
| 4 | 0.5M | 1 | 28 | 0 | 0 | 0 | 0 | 0 | 0 | 0.0 |
| 5 | 0.5M | 2 | 69 | 56 | 55 | 59 | 63 | 60 | 61 | 5.0 |
| 6 | 0.5M | 2 | 38 | 64 | 66 | 74 | 71 | 68 | 72 | 8.0 |
| 7 | 0.5M | 2 | 57 | 56 | 56 | 55 | 55 | 59 | 58 | 2.0 |
| 8 | 0.5M | 2 | 18 | 55 | 69 | 57 | 60 | 61 | 55 | 0.0 |
| 9 | 1.0M | 1 | 36 | 10 | 9 | 5 | 11 | 7 | 10 | 0.0 |
| 10 | 1.0M | 1 | 59 | 0 | 0 | 0 | 0 | 1 | 0 | 0.0 |
| 11 | 1.0M | 1 | 45 | 11 | 15 | 15 | 14 | 22 | 15 | 4.0 |
| 12 | 1.0M | 1 | 40 | 63 | 67 | 66 | 68 | 71 | 69 | 6.0 |
| 13 | 1.0M | 2 | 52 | 60 | 60 | 58 | 55 | 57 | 56 | -4.0 |
| 14 | 1.0M | 2 | 30 | 66 | 66 | 70 | 72 | 64 | 69 | 3.0 |
| 15 | 1.0M | 2 | 60 | 64 | 69 | 66 | 71 | 67 | 66 | 2.0 |
| 16 | 1.0M | 2 | 56 | 64 | 68 | 69 | 67 | 64 | 70 | 6.0 |
| 17 | 2.0M | 1 | 72 | 14 | 12 | 18 | 21 | 19 | 19 | 5.0 |
| 18 | 2.0M | 1 | 29 | 4 | 5 | 4 | 2 | 2 | 0 | -4.0 |
| 19 | 2.0M | 1 | 62 | 56 | 54 | 54 | 51 | 33 | 46 | -10.0 |
| 20 | 2.0M | 2 | 42 | 40 | 29 | 31 | 22 | 29 | 22 | -18.0 |
| 21 | 2.0M | 2 | 73 | 43 | 45 | 46 | 40 | 55 | 58 | 15.0 |
| 22 | 2.0M | 2 | 54 | 66 | 72 | 70 | 67 | 67 | 67 | 1.0 |
| 23 | 3.0M | 1 | 48 | 55 | 62 | 58 | 57 | 63 | 55 | 0.0 |
| 24 | 3.0M | 1 | 54 | 32 | 36 | 36 | 33 | 32 | 35 | 3.0 |
| 25 | 3.0M | 1 | 67 | 33 | 36 | 36 | 39 | 34 | 36 | 3.0 |
| 26 | 3.0M | 2 | 48 | 78 | 82 | 75 | 79 | 73 | 72 | -6.0 |
| 27 | 3.0M | 2 | 28 | 68 | 69 | 62 | 67 | 64 | 65 | -3.0 |
| 28 | 3.0M | 2 | 40 | 57 | 53 | 51 | 55 | 51 | 44 | -13.0 |
